# Establishing the acceptability of a brief patient reported outcome measure and feasibility of implementing it in a breast device registry – a qualitative study

**DOI:** 10.1186/s41687-019-0152-z

**Published:** 2019-10-22

**Authors:** Sze Ng, Maggie Kirkman, Jane Fisher, Andrea Pusic, Emily Parker, Rodney D. Cooter, Elisabeth Elder, Colin Moore, John McNeil, Ingrid Hopper

**Affiliations:** 1 Department of Epidemiology and Preventive Medicine, School of Public Health and Preventive Medicine, Monash University, Melbourne, VIC 3004 Australia; 2 Memorial Sloan-Kettering Cancer Center, New York, NY USA; 3 Australian Society of Plastic Surgeons, Sydney, NSW Australia; 4 Breast Surgeons of Australia and New Zealand, Randwick, NSW Australia; 5 Australasian College of Cosmetic Surgery, Parramatta, NSW Australia

**Keywords:** Patient reported outcome measures, Outcomes, Breast implant, Breast augmentation, Breast reconstruction, Acceptability, Feasibility

## Abstract

**Background:**

To examine the acceptability of a Patient Reported Outcome Measure (PROM) that assesses perceptions and experiences of implants for breast reconstruction or augmentation, and the feasibility of implementing it in the Australian Breast Device Registry (ABDR).

**Methods:**

The BREAST-Q Implant Surveillance (BREAST-Q IS) is a 5-question PROM derived from the BREAST-Q questionnaire. It assesses perceptions of breast appearance and sensation, and experiences of pain. Breast implant recipients (recruited via community networks, social media and notices in surgeons’ rooms) and surgeons contributing to the ABDR were invited to review the BREAST-Q-IS. Participation was by individual semi-structured interviews by telephone or email, or by completion of a paper questionnaire. Transcripts of audio recordings and emailed text were analysed thematically.

**Results:**

Twenty one breast implant recipients (10 after reconstruction and 11 augmentation), 8 surgeons (five plastic, three breast) and 2 medical professionals performing cosmetic surgeries were interviewed. Six themes were identified: *Overall impression*, *Emotional response to the BREAST-Q IS, Method of follow-up*, S*uggested improvements*, *Group variation,* and *Potential Clinical utility*. Overall, breast implant recipients and surgeons found the BREAST-Q IS to be acceptable and unlikely to provoke strong emotional reactions. Email was the preferred mode of contact. Most suggested improvements were to add questions. Surgeons expressed concern that subjective responses to the PROM might not accurately reflect experiences and that the PROM would predict need for revision rather than device failure.

**Conclusion:**

This study supports the acceptability and feasibility of BREAST-Q IS as a PROM for recipients of breast implants. Further validation of the Breast-Q IS is required.

## Introduction

Clinical quality registries (CQR) are organisations with the clearly defined purpose of improving the safety and quality (appropriateness and effectiveness) of health care by routinely and systematically collecting, analysing and reporting health-related information, within specific clinical domains [1]. CQR are recognised contributors to health systems that are able to provide timely, relevant and reliable feedback about patient care to clinicians, generate early warning of lowered outcomes, and serve as a means to share learning from high performing units [2].

A Patient Reported Outcome Measure (PROM) is defined as “*any report of the status of a patient’s health condition that comes directly from the patient, without interpretation of the patient’s response by a clinician or anyone else”* [3]. PROMs collection is central to continuous quality improvement processes and can change how healthcare is organised and delivered [4]. The impact of PROMs includes improving patient-provider communication [5], enhancing patients’ experience and satisfaction [6], and helping to identify unrecognised problems [6].

The purpose of using PROMs in a breast device registry is to provide early warning of underperforming devices via patient reports of breast device features at a time beyond regular surgical clinical follow-up. However, before implementing PROMs in a clinical quality registry, it is important to ensure the PROMs tool is acceptable. More importantly, the development of PROMs should be a collaborative effort with patients as partners in the research process, not solely as subjects, to ensure the relevance, acceptability, and quality of research [7].

The BREAST-Q Implant Surveillance (BREAST-Q IS) is a five-question PROM tool that was developed from the BREAST-Q for the purpose of assessing device performance. The BREAST-Q was developed by Pusic and her team through patient interviews, focus groups, expert panel and a literature review and has undergone thorough validation [8]. The BREAST-Q used after breast augmentation, breast reconstruction, and breast reduction surgery, has 88 questions across two domains (Quality of Life and Satisfaction) and six subdomains (Physical well-being, Psychosocial well-being, Sexual well-being, Satisfaction with breasts, Satisfaction with outcome, and Satisfaction with care) [8]. The BREAST-Q IS comprises five questions selected from the BREAST-Q sub-domains of Physical Well-being (within Quality of Life Domain) and Satisfaction with Breasts (within Satisfaction Domain). Analysis of more than 17,000 completed responses to the BREAST-Q survey identified the 5 items that make up the BREAST-Q IS as most sensitive to device issues performance and problems [9]. The BREAST-Q IS does not replace the BREAST-Q.

The aims of this research were to assess (i) the acceptability of the BREAST-Q IS to people who have breast implants, (ii) surgeons’ opinion of the BREAST-Q IS, (iii) preferred mode of contact for patients, and (iv) feasibility of the BREAST-Q IS for the ABDR.

## Methods

A qualitative method was appropriate because we sought to assess subjective responses, based on personal position and experience, to the BREAST-Q IS [10]. In establishing acceptability we relied on the common definition of ‘capable or worthy of being accepted’ [11], and similarly, on the meaning of feasibility as ‘the state or degree of being easily or conveniently done’ [12].

### Setting

The Australian Breast Device Registry (ABDR) is an Australian Government-funded clinical quality registry which commenced nationally in 2015. It collects surgical and device data at population level, aiming to include all patients having breast device procedures, all breast devices, and all surgeons performing these procedures across Australia [13]. Its goals are to drive evidence-based practice in breast device surgery and improve the quality of patient care [14, 15]. ABDR is governed by a steering committee which includes a management committee, membership of which includes clinical leads from three peak Australian surgical societies with interest in breast device monitoring and safety: Australian Society of Plastic Surgeons, Australasian College of Cosmetic Surgery, and Breast Surgeons of Australia and New Zealand Inc. [13]. The ABDR is a link among patients, surgeons, and various consumer advocacy groups. According to the ABDR’s 2017 Annual Report, there are 25,386 patients in the database [13].

### Participants

There were two groups of participants. The first group consisted of people who had received breast implants either for reconstruction (“Reconstruction”) or for cosmetic augmentation purposes (“Augmentation”). Breast implant recipients were eligible to participate if they were aged over 18 and able to communicate in English. We sought participants who were diverse in age, residence in metropolitan or rural areas, years since surgery, number of revisions, and whether surgery had been in Australia or overseas. We aimed to recruit about 20 participants, which in our experience would yield adequate data to assess views of the BREAST-Q IS for the breast device registry.

The second group was made up of plastic and reconstructive surgeons, breast surgeons, and medical practitioners performing cosmetic procedures, with various years of experience in breast implant surgery. We aimed to recruit about 10 practitioners to appraise the potential clinical utility of the BREAST-Q IS for the breast device registry.

### Recruitment

A purposive sampling strategy was used to recruit a heterogeneous sample of recipients of breast implants. To maximise diversity of backgrounds and experiences, consistent with qualitative research practice [10], various networks and modes of contact were used to invite volunteers. Networks included a women’s health consumer advocacy group which posted a flyer on its Facebook page and a breast cancer patient support network which emailed invitations to 426 women. An invitation was posted on ABDR social media accounts and four surgeons publicised the research at their clinics.

### Measures

Components of the BREAST-Q IS were selected carefully after repeated discussions by the authors of the BREAST-Q, the ABDR steering committee, and clinical leads. These items were considered to be most likely to predict implant performance and provide early warning of an underperforming device.

### Data source

We wanted to elicit opinions and enable participants to comment freely on the proposed PROM [10]. A study-specific interview guide (Additional file 2) was developed comprising demographic details and open-ended questions about the overall impression of the BREAST-Q IS as a means of assessing the condition of implants, relevance and acceptability of each question, and appropriate follow up strategy. It also invited suggestions for improving the PROM for use by the ABDR.

### Procedure

Breast implant recipients who volunteered were invited to participate in an individual telephone interview or to complete the survey by email. Paper-based questionnaires with reply-paid envelopes for return when completed were made available in the waiting rooms of four surgeons who agreed to advertise the survey. We offered several methods for completion for the convenience of participants and to increase the likelihood of completion.

Surgeons contributing to the ABDR were invited by personal email and through the ABDR newsletter to appraise the BREAST-Q IS and then participate in a 20-min telephone interview to provide feedback on its potential clinical utility as a PROM. Telephone interviews were chosen for surgeons because experience suggested that this was the most likely means of encouraging completion. The interview guide for breast device recipients was adapted for surgeons (Additional file 3).

All who agreed to participate were sent a copy of the BREAST-Q IS before the interview to allow them to reflect on it (Additional file 1). Recruitment commenced in October 2016 and ended in June 2017. It was made clear in the Explanatory Statement sent to all participants that the PROM was for use with people listed on the breast device registry.

Oral consent was audio-recorded at the beginning of telephone interviews. When participants chose to answer questions in writing, consent was implied. All telephone interviews (conducted by the first author) were audio-recorded and transcribed verbatim. Transcripts were de-identified before analysis. Alphanumeric codes were used to identify participants.

The research was approved by the Alfred Hospital Ethics Committee (459/16).

### Data analysis

Thematic analysis enabled us to learn about planned themes, evident in the interview guide, as well as unexpected themes encouraged by our open-ended questions. Analysis, using a well-established procedure [16], was conducted by SN and MK. Transcripts were first searched for themes arising from the questions asked of participants. Unexpected and unsought themes were identified by further close examination of the data. The iterative process involved close discussion between the two primary analysts and consideration of emerging results by the research team. Any differences were resolved by discussion.

## Results

### Recipients of breast implants

Twenty-one recipients of breast implants (10 reconstruction, 11 augmentation) volunteered and were interviewed (16 in writing, 5 by telephone). There were no discernible differences in responses that could be traced to mode of participation, except that written responses tended to be more succinct. Participants with reconstruction were older (most aged over 50 years) than those who had had augmentation (all aged under 50 years). Most lived in or within 2 hours from a major city. Surgery in the reconstruction group was performed in Australia; two of the augmentation group had surgery overseas. Silicone implants were used for all but one recipient. Half of both groups had undergone at least one revision. Table 1 shows characteristics of recipients of breast implants.

**Table 1:** Characteristics of recipients of breast implants

Characteristics	Breast reconstruction (*n* = 10)	Breast Augmentation (*n* = 11)
Age		
18–30	0	1
31–50	1	10
> 50	9	
Member of breast cancer support group		
Yes	6	0
No	4	11
Site of surgery		
Overseas	0	2
Australia	10	9
Years since implant surgery		
< 1 year	2	6
1–5 years	4	3
5–10 years	4	2
Place of residence		
In a major city	7	5
< 2 h from a major city	2	6
> 2 h from a major city	1	0
Number of implant revisions		
No revisions	5	6
1–3 revisions	3	4
> 3 revisions	2	1
Type of implant		
Tissue expander	1	0
Silicone	9	11
Non-silicone	0	0

### Surgeons

Ten surgeons volunteered and were interviewed by telephone. Six performed more than one type of breast surgery (reconstruction, augmentation, tissue expander), five had performed breast device surgery as independent practitioners for at least 15 years, eight had been practising during the Poly Implant Prostheses incident (in which non-medical grade silicone was used in breast implants which was purported to be have an increased rate of implant rupture) [17], five performed more than 50 device surgeries annually and five performed fewer, and four stated that they were investigators on current or past breast implant research.

### Themes

In addition to themes established by the questions asked of participants (*Overall acceptability of BREAST-Q IS*, *Emotional acceptability of the BREAST-Q IS*, *Feasibility and mode of contact*, and *Improvements*), the themes of *Group variation* and *Potential clinical utility* were identified. Themes are described below with illustrative quotations (R = Reconstruction, A = Augmentation, S = Surgeon).

#### Striking the balance between data burden vs data meaningfulness

Overall, recipients and surgeons found the BREAST-Q IS to be acceptable. The discussions related to striking the balance between data burden and data meaningfulness. Most of them described the BREAST-Q IS in positive terms such as a “*good idea*” (ID01A, ID03A, ID04A), and “*ideal and the most important questions*” (ID09S). There was surprise at its brevity, reflected in the comment that “*more data needs to be collected*” (ID01R). However, there was also appreciation of the brevity, such as “*it’s good that the questionnaire is brief*” (ID04R). One surgeon rejected the BREAST-Q IS because it would produce “*a lot of noise*” (ID08S), implying that responses would not be relevant to surgeons.

#### Emotional acceptability

Recipients and surgeons were asked whether they thought the questions would upset, annoy or trouble a person with breast implants. On the whole, neither group envisaged an adverse emotional response, although one recipient thought that it “*may draw attention to/reinforce any dissatisfaction with the outcome, as well as reminding the person they had implants because they had cancer*” (ID10R).

#### Feasibility and mode of contact

Recipients thought that it was acceptable and feasible to receive follow up from ABDR. Of the modes of contact proposed (text message, telephone, email, or post), the majority of recipients nominated email as their preferred mode of contact. One recipient of reconstruction said that, for communication “*about impersonal things, I am much more resistant to engage in a text message*” (ID09R). Surgeons also preferred email as mode of contact for patients in their care.

#### Improvements

Where improvements to the BREAST-Q IS were suggested, they usually concerned adding more questions. Recipients proposed questions about the experience of receiving a breast implant. These included “*overall satisfaction*” (ID09A), “*changes to or restrictions in movement*” (ID02R, ID03R), “*whether the clothes fit right*” (ID05R), and implants as a “*barrier to intimacy*” (ID09R). Suggested questions about satisfaction included, “Have the implants satisfied the reasons for getting them?” (ID03A), “If there is one thing you could change about your breast implant, what would it be?” (ID10R), and “Have you sought advice for issues with pain or tightness experienced?” (ID01R). Surgeons were more likely to propose adding questions about clinical outcome. They wanted to know about satisfaction with shape and symmetry, especially after reconstruction (ID01S), with one explaining that “*they may sort of be satisfied with the shape of the breast but they are very unhappy about being asymmetrical*” (ID04S). Whether the implant had noticeably changed was thought to be important by two surgeons (ID04S, ID08S); for example, “*Has the breast become softer when it was previously hard or it has become hard when it was previously softer?*” (ID04S) and “*Do you feel your breasts have dropped?*” (ID08S).

#### Group variation

There were differences in the concerns identified as important by augmentation and reconstruction recipients. Augmentation recipients were aware, for example, of the influence of weight loss or gain on “*rippling*” (ID01A, ID10A) and that the feel of breast to touch and breast pain can vary depending on the stage in the menstrual cycle (ID09A). Reconstruction recipients wanted to be able to explain how and why they were dissatisfied with their breasts (ID01R, ID02R, ID04R), including experiences of lack of sensation (ID09R) and the adequacy of the information they received (ID01R, ID06R).

#### Potential clinical utility

Surgeons expressed mixed opinions about using BREAST-Q IS to assess outcomes of breast augmentation and reconstruction. There was support for subjective reports from patients: “*One of the powerful things about the Breast Q is that it is coming from a patient perspective and not a clinician perspective*.” (ID06S) However, concerns were also stated about using the BREAST-Q IS. Some surgeons thought that recipients’ subjective responses amounted to “*noise*” (ID08S), with one doubting that “*you can make any sense out of that information*” (ID03S). There were disagreements about the choice of questions. For example, it was said that recipients should not be asked about unclothed appearance because that would “*bias the outcomes* ... *as the typical goal is actually to give them a shape that they are happy with whilst they are clothed”* (ID10S). There was also implicit concern about reputational damage from the BREAST-Q IS arising from unjust criticism of surgical outcome based on breast implant recipients’ satisfaction. One surgeon said that what would objectively be a good clinical outcome (successful surgery) might not satisfy the recipient: “*This is an extremely subjective process, what women feel about the shape of their breast whereby their breast might not have changed shape at all*” (ID08S).

Surgeons thought that the BREAST-Q IS could predict the risk of implant revision surgery because recipients who are dissatisfied with their breast implant will consider revision surgery, but that the BREAST-Q IS has limited potential clinical utility for predicting device failure. One surgeon provided a detailed explanation: “*If somebody is dissatisfied with their breast augmentation or their reconstruction, it might have been that the choice of the implant wasn’t the best choice for the patient. ... Or the patient’s changed their mind about what size they want to be. … There is nothing wrong with the implant”* (ID04S). Surgeons commented on how the BREAST-Q IS questions might help with predicting risk of implant revision: “*There will be a positive correlation between satisfaction and revision; … the unhappy patients are the ones that are going to get revised*” (ID06S).

## Discussion

For this study, we interviewed a heterogeneous sample of breast implant recipients and surgeons, finding the BREAST-Q IS to be acceptable and feasible as a PROM for recipients of breast implants. This was not a prevalence study; matching a ‘sample’ to the population was unnecessary. Had there been any evidence that the instrument was unacceptable or infeasible to administer, we would have amended the instrument and repeated the process. Results were sufficient to encourage us to proceed with using the instrument. This was the aim of the investigation.

Common patient-related barriers to PROM completion include perception that a questionnaire is too long and that the questions are irrelevant or confusing [18]. The Breast Q IS was designed to be short and easy to understand. Although the brevity of the BREAST-Q IS was seen as a strength by most recipients and surgeons, some participants suggested further questions in pursuit of matters that they considered to be important. Augmentation recipients offered relatively few comments about the PROM in this study, suggesting that this group might be less likely to engage with a longer survey. Reconstruction recipients were keen to relate their experiences in detail. This suggests that there could be opportunities for further qualitative research about breast reconstruction experiences. Although additional questions were suggested as potential improvements, we submit that these may be more suitable for targeted investigation using qualitative methods rather than for a succinct PROM focused on device performance.

We expect that matters raised in this study about the effects of weight change and the menstrual cycle will not disrupt the ability of the Breast-Q IS to signal an under-performing device, as all devices will be equally affected. With increased patient engagement and shared decision-making, these meaningful data will contribute to better understanding of expected outcomes [19]. This is a perfect opportunity, as patients have been found to be more engaged in their healthcare [20]. The Breast-Q IS can be incorporated into an existing data set and may serve in a composite quality measure in conjunction with existing clinical data [14]. Indeed, PROMs have been selected as quality indicators for use by the ABDR and international breast device registries.

It was evident that some surgeons we interviewed found the use of subjective information from patients challenging. This is consistent with a recent systematic review in which physician resistance to PROMs was identified [21]. Reasons for resistance were similar to those found in our study: difficulty in interpreting PROM data and doubt about whether PROM data genuinely reflect care [22, 23]. It is important to note that PROMs are designed to capture patients’ subjective experiences and that they are increasingly used to evaluate therapies [24]. As uptake of PROMs by medical specialties increases, we expect this concern to be allayed [5]. The capability of the BREAST-Q IS to identify device safety and underperformance will also be addressed as the ABDR collects more data, and results can be correlated with revision rates as well as the broader potential for data linkage with other breast device registries worldwide.

There are practical matters that must be addressed when undertaking national roll-out of the BREAST-Q IS First is confidentiality of patients and surgeons. Aggregate results will be reported, and all comments will be confidential with de-identified data. However, clinicians will be able to view their own aggregate results confidentially and compare them to a population mean, which has the potential to drive quality improvement. The method of contact is also crucial. Email addresses are not routinely collected on the hospital identification label; we have email addresses only for 10% of those on the ABDR, but mobile telephone numbers for 70%. Contact by text message was also found acceptable as a method of invitation to complete the survey online in this study. According to the Mobile Consumer Survey 2016, smartphone ownership in Australia has been rising steadily from 76% in 2014 to 84% in 2016 [25]. It was recently found that there is no difference in response rates between online and paper questionnaire invitational methods among older people treated for colorectal cancers [26]. This suggests that our response rate for our future national follow-up roll-out will not be reduced if web-based invitations are used for older patients, especially in the breast reconstruction cohort. A few participants were concerned that, although the Breast-Q IS questions are not in themselves distressing, the topic may be emotionally arousing. We have therefore developed a comprehensive training manual to ensure that study staff can offer contact details to any patient wanting to consult a mental health professional.

### Study limitations

Surgeons who participated in this study all support and contribute data to the ABDR. It is possible that surgeons not sharing these characteristics might have different views. However, we purposively selected practitioners to represent surgeons who perform breast implant surgeries in Australia, including from all three craft groups, with a range of years in practice in different practice locations. The concerns expressed by some surgeons about PROMs lead us to propose engaging professionals (as well as patients) in the planning stage of a PROM and ensuring high levels of transparency in the rationale of data collection. This insight indicates that the surgeons have contributed to our understanding of the BREAST-Q IS and its application.

The use of an appropriate qualitative method to capture diverse perspectives on a topic cannot be considered a limitation. The value of such research lies in gaining understanding of what a topic *means* to participants, not how many share a particular view. Our results tell us, therefore, of some of the positions adopted by breast device recipients and surgeons but lay no claim to being comprehensive nor proportional to what may be found in the population. Where meaning and perspective are concerned, it can usually be assumed that each person will be at least subtly different. We purposively recruited participants with diverse characteristics and experiences of breast implant surgery, but others will have different experiences and perspectives. Should other registries seek to make use of the BREAST-Q IS, it could be useful to conduct a similar investigation with people selected from local populations.

## Conclusion

This study has assessed the acceptability and feasibility of using the BREAST-Q IS as a PROM tool, and found it has generally good acceptability for breast implant recipients and surgeons. Its brevity means that it is feasible to use on a large scale. Using the BREAST-Q IS consistently may enable us to identify changes that predict the need for revision surgery, thus providing an early signal of under-performing devices. The ABDR will perform long-term patient follow up at one, two, five, and ten years after their surgery to detect poor device performance and safety beyond routine clinical follow up. Further work is required to validate the BREAST-Q IS, which will be done by comparing data from the BREAST-Q, test-retest reliability study and to determine its efficacy as a tool for the long-term monitoring of device safety.

## Supplementary information

**Additional file 1.** BREAST-Q Implant Surveillance (BREAST-Q IS)

**Additional file 2.** Questions for Women (Interview) BreastAug and BreastRec

**Additional file 3.** Questions for Surgeons (BreastAug and BreastRec)

## Data Availability

The dataset analyzed during the current study is available from the corresponding author on reasonable request.

## Abbreviations

ABDR: Australian Breast Device Registry
BREAST-Q IS: BREAST-Q Implant Surveillance
CQR: Clinical quality registries
PROM: Patient Reported Outcome Measure

## References

[1] Australian Commission on Safety and Quality in Health Care (2014). *Framework for Australian clinical quality registries*.

[2] Wilcox N, McNeil JJ (2016). Clinical quality registries have the potential to drive improvements in the appropriateness of care. The Medical Journal of Australia.

[3] Administration USDoHaHSFaD (2009). *Guidance for industry patient-reported outcome measures: Use in medical product development to support labeling claims*.

[4] Black N. (2013). Patient reported outcome measures could help transform healthcare. BMJ.

[5] Rotenstein LS, Huckman RS, Wagle NW (2017). Making patients and doctors happier - the potential of patient-reported outcomes. The New England Journal of Medicine.

[6] Chen, J., Ou, L., & Hollis, S. J. (2013). A systematic review of the impact of routine collection of patient reported outcome measures on patients, providers and health organisations in an oncologic setting. *BMC Health Services Research, 13*(211). https://www.ncbi.nlm.nih.gov/pubmed/?term=A+systematic+review+of+the+impact+of+routine+collection+of+patient+reported+outcome+measures+on+patients%2C+providers+and+health+organisations+in+an+oncologic+setting.+BMC+Health+Services+Research%2C+13(211). 10.1186/1472-6963-13-211 PMC3700832 23758898

[7] Staniszewska S, Haywood KL, Brett J, Tutton L (2012). Patient and public involvement in patient-reported outcome measures: Evolution not revolution. Patient.

[8] Pusic AL, Klassen AF, Scott AM (2009). Development of a new patient-reported outcome measure for breast surgery: The BREAST-Q. Plastic and Reconstructive Surgery.

[9] Ng, S., Pusic, A., Parker, E., et al. (2019). Patient-reported outcome measures for breast implant surgery: A pilot study. *Aesthetic Surgery Journal*. 10.1093/asj/sjz023. https://www.ncbi.nlm.nih.gov/pubmed/30783646 10.1093/asj/sjz023 30783646

[10] Hammarberg K, Kirkman M, de Lacey S (2016). Qualitative research methods: When to use them and how to judge them. Human Reproduction.

[11] Macquarie Dictionary (2018) https://www.macquariedictionary.com.au/. Accessed 12 Oct 2018.

[12] Oxford Dictionaries (2019) https://en.oxforddictionaries.com/definition/acceptability. Accessed 3 May 2019.

[13] ABDR (2019). *The Australian breast device registry 2017 report*.

[14] Hopper I, Ahern S, Best RL, McNeil J, Cooter RD (2017). Australian breast device registry: Breast device safety transformed. ANZ Journal of Surgery.

[15] McNeil JJ, Evans SM, Johnson NP, Cameron PA (2010). Clinical-quality registries: Their role in quality improvement. The Medical Journal of Australia.

[16] Braun V, Clarke V (2006). Using thematic analysis in psychology. Qualitative Research in Psychology.

[17] Australia Government DoH (2010). *Therapeutic goods administration silicone gel breast implants manufactured by poly implant Prothese (PIP) of France*.

[18] Franklin PD, Lewallen D, Bozic K (2014). Implementation of patient-reported outcome measures in U.S. Total joint replacement registries: Rationale, status, and plans. The Journal of Bone and Joint Surgery. American Volume.

[19] Pusic AL, Reavey PL, Klassen AF (2009). Measuring patient outcomes in breast augmentation: Introducing the BREAST-Q augmentation module. Clinics in Plastic Surgery.

[20] 2016 CDW Healthcare Patient Engagement Study. https://cdw-prod.adobecqms.net/content/dam/cdw/on-domain-cdw/cdw-branded/newsroom/tech-insights/February-2016-Patient-Engagement-Key-Findings-Study.pdf. Accessed 15 Oct 2019.

[21] Boyce MB, Browne JP, Greenhalgh J (2014). The experiences of professionals with using information from patient-reported outcome measures to improve the quality of healthcare: A systematic review of qualitative research. BMJ Quality and Safety.

[22] Callaly T, Hyland M, Coombs T, Trauer T (2006). Routine outcome measurement in public mental health: Results of a clinician survey. Australian Health Review.

[23] Mitchell C, Dwyer R, Hagan T, Mathers N (2011). Impact of the QOF and the NICE guideline in the diagnosis and management of depression: A qualitative study. The British Journal of General Practice.

[24] Frosch DL (2015). Patient-reported outcomes as a measure of healthcare quality. Journal of General Internal Medicine.

[25] Deloitte (2016). *Mobile consumer survey 2016: The Australian cut*.

[26] Horevoorts NJ, Vissers PA, Mols F, Thong MS, van de Poll-Franse LV (2015). Response rates for patient-reported outcomes using web-based versus paper questionnaires: Comparison of two invitational methods in older colorectal cancer patients. Journal of Medical Internet Research.

