# Supplementary material for: Establishing the acceptability of a brief patient reported outcome measure and feasibility of implementing it in a breast device registry – a qualitative study
Source: J Patient Rep Outcomes. 2019 Oct 22;3:63. doi: 10.1186/s41687-019-0152-z (PMC6805841; doi:10.1186/s41687-019-0152-z)
Supplement: Supplementary file 1 — Additional file 1. BREAST-Q Implant Surveillance (BREAST-Q IS) [file 41687_2019_152_MOESM1_ESM.docx]

With your breasts in mind, in the past week, how satisfied or dissatisfied have you been with:

|  | **Very Dissatisfied** | **Somewhat Dissatisfied** | **Somewhat Satisfied** | **Very Satisfied** |
| --- | --- | --- | --- | --- |
| a. The shape of your breasts when you are not wearing a bra? | 1 | 2 | 3 | 4 |
| b. How your breasts feels to touch? | 1 | 2 | 3 | 4 |
| c. The amount of rippling (wrinkling) of your implant(s) that you can see? | 1 | 2 | 3 | 4 |

In the past week, how often have you experienced:

|  | **None of the time** | **A little of the time** | **Some of the time** | **Most of the time** | **All of the time** |
| --- | --- | --- | --- | --- | --- |
| a. Pain in your breast area? | 1 | 2 | 3 | 4 | 5 |
| b. Tightness in your breast area? | 1 | 2 | 3 | 4 | 5 |

With your breasts in mind, in the past week, how satisfied or dissatisfied have you been with:

|  | **Very Dissatisfied** | **Somewhat Dissatisfied** | **Somewhat Satisfied** | **Very Satisfied** |
| --- | --- | --- | --- | --- |
| a. The shape of your reconstructed breast(s) when you are not wearing a bra? | 1 | 2 | 3 | 4 |
| b. How your reconstructed breast(s) feels to touch? | 1 | 2 | 3 | 4 |
| c. The amount of rippling (wrinkling) of your implant(s) that you can see? | 1 | 2 | 3 | 4 |

In the past week, how often have you experienced:

|  | **None of the time** | **A little of the time** | **Some of the time** | **Most of the time** | **All of the time** |
| --- | --- | --- | --- | --- | --- |
| d. Pain in your reconstructed breast(s) area? | 1 | 2 | 3 | 4 | 5 |
| e. Tightness in your reconstructed breast(s) area? | 1 | 2 | 3 | 4 | 5 |
