# Supplementary material for: Establishing the acceptability of a brief patient reported outcome measure and feasibility of implementing it in a breast device registry – a qualitative study
Source: J Patient Rep Outcomes. 2019 Oct 22;3:63. doi: 10.1186/s41687-019-0152-z (PMC6805841; doi:10.1186/s41687-019-0152-z)
Supplement: Supplementary file 2 — Additional file 2. Questions for Women (Interview) BreastAug and BreastRec [file 41687_2019_152_MOESM2_ESM.docx]

The Australian Breast Device Registry (ABDR) has been set up to ensure that breast implant devices are safe for those who use them. We think the registry can be most useful if we contact registered women from time to time to check on the condition of their breast implants. We’ve drafted a set of questions (called the BREAST-Q IS) to use when we contact women, and would be grateful for your help in improving them. We also want to learn what you think is the best way to contact women to invite them to answer these questions.

Have you had a chance to read the information sheet that I sent to you? Would you like to ask any questions? May I record our conversation?

Would you please state formally that you consent to participating in this research interview? Thank you.

# First, we’d like to know what you think of the BREAST-Q IS: the questions for women who have breast implants

Please look at the BREAST-Q IS document. If you haven’t had a chance to read it, you might like to read it now. [*Allow time to read it if necessary*.]

Do you have any comments to make about it in general?

Now I’d like you to comment on each of the questions in turn.

***(Depending on type of breast surgery the woman had, we ask the relevant sections of the question)***

**BREAST AUGMENTATION**

**The first questions are for women who have implants for breast AUGMENTATION or breast enlargement. They ask how satisfied or dissatisfied each woman has been in the past week with**

1. The shape of her breasts when she is not wearing a bra.
2. How her breasts feel to the touch. And
3. The amount of rippling (wrinkling) of her implants that she can see.

- Do you think these questions will be relevant to a woman with implants for breast enlargement?

Yes ❑ No ❑ Mixed ❑ I don’t know ❑ *[Don’t read these responses; they are for the interviewer’s use only]*

*[If necessary, ask her to expand on her answer using an appropriate form of words; the following is just a suggestion:]* Can you tell me a bit more about why you think this way?

- Do you think these questions might upset, or annoy, or otherwise trouble a woman with implants for breast enlargement?

Yes ❑ No ❑ Mixed ❑ I don’t know ❑ *[Don’t read these responses; they are for the interviewer’s use only]*

*[If necessary, ask her to expand on her answer using an appropriate form of words; the following is just a suggestion:]* Why do you think they will be troubling/annoying/upsetting?

- Can you suggest better ways to ask about satisfaction with implants for breast enlargement?

# The second set of questions for women who’ve had breast augmentation or enlargement, we ask each woman how often she’s experienced two conditions in the past week:

1. Pain in her breast area, and
2. Tightness in her breast area.

- Do you think these questions will be relevant to a woman with implants for breast enlargement?

Yes ❑ No ❑ Mixed ❑ I don’t know ❑ *[Don’t read these responses; they are for the interviewer’s use only]*

*[If necessary, ask her to expand on her answer using an appropriate form of words; the following is just a suggestion:]* Can you tell me a bit more about why you think this way?

- Do you think these questions might upset, or annoy, or otherwise trouble a woman with implants for breast enlargement?

Yes ❑ No ❑ Mixed ❑ I don’t know ❑ *[Don’t read these responses; they are for the interviewer’s use only]*

*[If necessary, ask her to expand on her answer using an appropriate form of words; the following is just a suggestion:]* Why do you think they will be troubling/annoying/upsetting?

- Can you suggest better ways to ask a woman with implants for breast enlargement about these conditions?

# BREAST RECONSTRUCTION

**The first questions are for women who have implants for breast RECONSTRUCTION. They ask how satisfied or dissatisfied each woman has been in the past week with**

- 1. The shape of her reconstructed breast(s) when she is not wearing a bra.
  2. How her reconstructed breast feels to the touch. And
  3. The amount of rippling or wrinkling of her implant that she can see.
- Do you think these questions will be relevant to a woman with an implant for breast reconstruction?

Yes ❑ No ❑ Mixed ❑ I don’t know ❑ *[Don’t read these responses; they are for the interviewer’s use only]*

*[If necessary, ask her to expand on her answer using an appropriate form of words; the following is just a suggestion:]* Can you tell me a bit more about why you think this way?

- Do you think these questions might upset, or annoy, or otherwise trouble a woman with an implant for breast reconstruction?

Yes ❑ No ❑ Mixed ❑ I don’t know ❑ *[Don’t read these responses; they are for the interviewer’s use only]*

*[If necessary, ask her to expand on her answer using an appropriate form of words; the following is just a suggestion:]* Why do you think they will be troubling/annoying/upsetting?

- Can you suggest better ways to ask about satisfaction with an implant for breast reconstruction?

# The second set of questions for women who’ve had breast reconstruction, we ask each woman how often she’s experienced two conditions in the past week:

- 1. Pain in her breast area, and
  2. Tightness in her breast area.
- Do you think these questions will be relevant to a woman with implants for breast reconstruction?

Yes ❑ No ❑ Mixed ❑ I don’t know ❑ *[Don’t read these responses; they are for the interviewer’s use only]*

*[If necessary, ask her to expand on her answer using an appropriate form of words; the following is just a suggestion:]* Can you tell me a bit more about why you think this way?

- Do you think these questions might upset, or annoy, or otherwise trouble a woman with breast reconstruction?

Yes ❑ No ❑ Mixed ❑ I don’t know ❑ *[Don’t read these responses; they are for the interviewer’s use only]*

*[If necessary, ask her to expand on her answer using an appropriate form of words; the following is just a suggestion:]* Why do you think they will be troubling/annoying/upsetting?

- Can you suggest better ways to ask a woman with implants for breast reconstruction about these conditions?

# I’d now like to ask you about what influence these questions might have on women. One of our goals is to prompt women to take action about difficulties with their implants, if it’s necessary. Do you think our questions might encourage women to

- Seek support from a community group?

Yes ❑ No ❑ Mixed ❑ I don’t know ❑

- Seek advice from doctor?

Yes ❑ No ❑ Mixed ❑ I don’t know ❑

- Look up more information?

Yes ❑ No ❑ Mixed ❑ I don’t know ❑

*[Don’t read these responses; they are for the interviewer’s use only]*

*[If necessary, ask her to expand on her answer using an appropriate form of words; the following is just a suggestion:]* Can you tell me a bit more about why you think this way?

# I’m wondering whether you think there’s anything we need to add to the BREAST-Q IS. We don’t want to take up too much of your time, but we also don’t want to miss something important. Do you have any suggestions for something else we need to ask? *[If there is, please ask her to say why.]*

**I don’t have any more questions about the BREAST-Q IS, but I hope you’ll also be willing to answer four brief questions about being contacted by the Australian Breast Device Registry.**

These questions ask about your own preferences: for yourself, not women in general.

1. First, would you be willing to be contacted by the registry to update our records on your satisfaction and experience with your implant? Each time we’d be using the revised version of our BREAST-Q IS.

Yes ❑ No ❑ *[Don’t read these responses; they are for the interviewer’s use only] [If she says Yes:]* Thank you. *[Ask her the next question.]*

*[If she says No:]* Would you mind telling me why? *[Skip the next question]*

1. *[If she is willing to be contacted by the ABDR]* Can you tell me, please, how you’d prefer to be contacted? We could use text messaging, email, telephone, or ordinary mail.

- Text Messaging
- Email
- Telephone
- Mail

1. Below is an example of a text message that we would like to use to follow up women in the registry.

“ Hi , this is the ABDR team from Monash University (a government quality & safety initiative). As part of registry follow up process we need your help to answer 5 brief questions provided in the link below . More information about us at [www.abdr.org.au](http://www.abdr.org.au/) Please do not reply by sms.”

Do you think this text message is acceptable? Please provide any suggestion.

1. Is there anything else you would like to tell us?

We plan to invite women to participate in a future survey on breast implants. May we send you an invitation? It won’t put you under any obligation to accept.

Yes ❑ Best contact: No ❑

*[Don’t read these responses; they are for the interviewer’s use only]*

Thank you very much for answering all these questions. It’s been really helpful.

# FINALLY, I’d like to ask you a few questions about yourself so that we can summarise information about all the women who are helping us. These details will remain confidential; we won’t reveal anything about you personally.

***[Move to demographics document]***
