# Supplementary material for: Establishing the acceptability of a brief patient reported outcome measure and feasibility of implementing it in a breast device registry – a qualitative study
Source: J Patient Rep Outcomes. 2019 Oct 22;3:63. doi: 10.1186/s41687-019-0152-z (PMC6805841; doi:10.1186/s41687-019-0152-z)
Supplement: Supplementary file 3 — Additional file 3. Questions for Surgeons (BreastAug and BreastRec) [file 41687_2019_152_MOESM3_ESM.docx]

As you know, we would value your expert opinion on our draft PROMs tool, called the BREAST-Q Implant Surveillance (BREAST-Q IS). I hope you’ve had a chance to look at it. [*If not, allow time to read it*.]

I’m also going to ask your opinion about how we can best contact women on the registry as part of follow up.

May I record our conversation?

Would you please state formally that you consent to participating in this research interview? Thank you.

## First, we’d like to know what you think of the BREAST-Q IS

Please give me your overall impression of the document.

Now I’d like to ask you to comment on each of the questions in turn.

## The first questions are about AUGMENTATION, and ask the woman how satisfied or dissatisfied in the past week, she has been with

1. The shape of her breasts when she is not wearing a bra.
2. How her breasts feel to the touch.
3. The amount of rippling (wrinkling) of her implants that she can see.

- Do you think these questions are useful? Yes ❑ No ❑ Mixed ❑
- Can you suggest better ways to ask about satisfaction with augmentation?

## The second augmentation questions ask the woman how often she has experienced two conditions in the past week:

1. Pain in her breast area.
2. Tightness in her breast area.

- Do you think these questions are useful? Yes ❑ No ❑ Mixed ❑
- Can you suggest better ways to ask about these conditions?

## The second set of questions are about RECONSTRUCTION, similarly asking the woman how satisfied or dissatisfied in the past week, she has been with

- 1. The shape of her breasts when she is not wearing a bra.
  2. How her breasts feel to the touch.
  3. The amount of rippling (wrinkling) of her implants that she can see.
- Do you think these questions are useful? Yes ❑ No ❑ Some are ❑
- Can you suggest better ways to ask about satisfaction with reconstruction?

## The final reconstruction questions ask the woman how often she has experienced two conditions in the past week:

- 1. Pain in her breast area.
  2. Tightness in her breast area.
- Do you think these questions are useful? Yes ❑ No ❑ Some are ❑
- Can you suggest better ways to ask about these conditions?

I’ve asked about the usefulness of these questions. Now I’d like your opinion on **whether any of the questions are likely to make women anxious or irritated or to respond in any other undesirable way**.

If so, how can we improve them?

## One of our main goals is to use BREAST-Q IS to predict breast device failure. Do you think we can do so with these questions?

If not, do you think we can use the BREAST-Q IS for predicting risk of breast implants revision?

## We would like to follow up women in the registry. What do you think is the best method for contacting your type of patients?

(Text message….[list methods here in case a prompt is necessary]) (Frequency?)

Do you have any general comments about contacting women for follow-up?

## Finally, can you tell us your opinion on whether the BREAST-Q IS will contribute to improving patient safety?

If so, which part of the BREAST-Q IS would be most valuable to you? In what way?

## Is there anything else you’d like to say about the BREAST-Q IS?

**Thank you very much for helping us to refine the BREAST-Q IS.**

**Finally, I’d like to ask you a few questions about your specialty so that we can summarise details of the surgeons who had contributed to this project. *[DEMOGRAPHICS]***

Questions for Surgeons – Breast Aug Breast Recon (Version 2 dated 27 October2016)

## Demographics Questionnaire for Surgeons

Participant ID NO | | | | | Researcher Initials | | | | Date | | / | / | |

We need to be able to summarise some details about the surgeons who are contributing to this research, so we hope you will be prepared to answer a few questions about your professional background. *[If the Intro at the end of the BREAST-Q IS has been used, no need to repeat this.]*

1. How many years have you been performing breast device surgery independently (ie. without supervision)
   - Less than 5 years
   - 5-10 years
   - 10-15 years
   - More than 15 years
2. Were you practising during the PIP crisis in 2010?
   - Yes
   - No
3. How many breast device surgeries do you perform in a year?
   - Fewer than 10

❑ 10-50

- - More than 50

1. Are you an investigator on any current or past breast implant research?
   - Yes
   - No

Thank you. I don’t have any more questions.

Are there any questions you have about this project?

# Thank you for being so generous with your time and expertise.

Demographics Questionnaire for Surgeons (Version 1 dated 1 Sept2016)
